# 1,2,3-Triphenyl-1,2-dihydro­quinoxaline

**DOI:** 10.1107/S1600536808029905

**Published:** 2008-09-20

**Authors:** Frank T. Edelmann, Steffen Blaurock, Volker Lorenz, Axel Fischer

**Affiliations:** a Chemisches Institut, Otto-von-Guericke-Universität Magdeburg, Universitätsplatz 2, D-39106 Magdeburg, Germany

## Abstract

The title compound, C_26_H_20_N_2_, first reported in 1891, was obtained as a by-product in the preparation of benzildianil from benzil and excess aniline. The dihedral angles between the fused benzene ring and the pendant phenyl rings are 17.93 (11), 53.18 (10) and 89.08 (12)°.

## Related literature

For related literature, see: Bodforss (1960 ▶); Kehrmann & Messinger (1891 ▶); Sannicolò (1983 ▶); Lorenz *et al.* (1994 ▶); Siegfeld (1892 ▶).
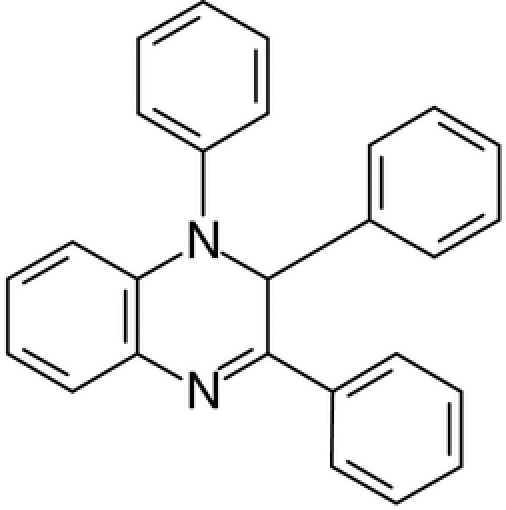


## Experimental

### 

#### Crystal data

C_26_H_20_N_2_
*M* *_r_* = 360.44
Monoclinic, 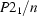

*a* = 10.121 (2) Å
*b* = 10.374 (2) Å
*c* = 18.572 (4) Å
β = 96.49 (3)°
*V* = 1937.4 (7) Å^3^
*Z* = 4
Mo *K*α radiation
μ = 0.07 mm^−1^
*T* = 143 (2) K
0.40 × 0.25 × 0.20 mm

#### Data collection

Stoe STADI4 diffractometer
Absorption correction: none
5587 measured reflections
3413 independent reflections
2249 reflections with *I* > 2σ(*I*)
*R*_int_ = 0.038
3 standard reflections frequency: 90 min intensity decay: none

#### Refinement

*R*[*F* ^2^ > 2σ(*F* ^2^)] = 0.053
*wR*(*F* ^2^) = 0.120
*S* = 1.06
3413 reflections
253 parameters
H-atom parameters constrained
Δρ_max_ = 0.17 e Å^−3^
Δρ_min_ = −0.20 e Å^−3^

### 

Data collection: *DIF4* (Stoe & Cie, 1992 ▶); cell refinement: *DIF4*; data reduction: *REDU4* (Stoe & Cie, 1992 ▶); program(s) used to solve structure: *SHELXS86* (Sheldrick, 2008 ▶); program(s) used to refine structure: *SHELXL97* (Sheldrick, 2008 ▶); molecular graphics: *XP5* in *SHELXTL* (Sheldrick, 2008 ▶); software used to prepare material for publication: *SHELXL97*.

## Supplementary Material

Crystal structure: contains datablocks I, global. DOI: 10.1107/S1600536808029905/bt2784sup1.cif

Structure factors: contains datablocks I. DOI: 10.1107/S1600536808029905/bt2784Isup2.hkl

Additional supplementary materials:  crystallographic information; 3D view; checkCIF report

## Figures and Tables

**Fig. 1.**
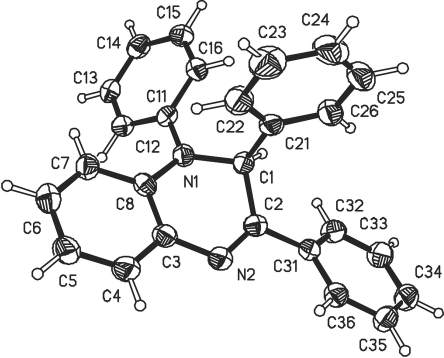
The molecule of the title compound in the crystal. Displacement ellipsoids represent 50% probability levels. H-Atom radii are arbitrary.

## supplementary crystallographic information

### Comment

The title compound, 1,2,4-triphenyl-1,2-dihydroquinoxaline (1), was first reported as early as 1891 by Kehrmann & Messinger. In the original paper the compound was named `quinoxaline base from benzoin and phenylphenylenediamine' and was already assigned the correct structure. It was described as `magnificent uranium-yellow crystals' showing a bright blue–green fluorescence in solution (alcohols, ether, benzene) (Kehrmann & Messinger, 1891). In 1960, Bodforss used 1 and substituted derivatives thereof to prepare cationic quinoxalinium salts which in many respects resembled the well known triphenylmethane dyes (Bodforss, 1960). For example, the dimethylamino derivatives were found to give blue solutions, while the dihydroxyphenyl derivative was orange–red. The only other report on 1 found in the literature is a paper by Sannicolò entitled `New Heterocyclic Syntheses from Benzil Dianils' (Sannicolò , 1983). In this paper it was reported that 1 is formed as a by-product in the preparation of benzildianil from benzil and excess aniline in the presence of 4-toluenesulfonic acid as catalyst. Under suitable reaction conditions the isolated yield of 1 can be as high as 31% (Sannicolò, 1983). We obtained the beautiful yellow crystals of the title compound by chance in minor amounts as a by-product in the preparation of benzildianil according to the literature procedure (Siegfied *et al.*, 1892; Lorenz *et al.*, 1994). A crystal structure determination of revealed the presence of 1,2,4-triphenyl-1,2-dihydroquinoxaline (1) containing three adjacent phenyl substituents attached to the 1,2-dihydroquinoxaline core. With a short distance of 1.282 (3) Å the C2—N2 bond is clearly a double bond. Thus this study confirmed that Kehrmann & Messinger had assigned the correct structure of the title compound almost 120 years ago (Kehrmann & Messinger, 1891).

### Experimental

Well formed, bright yellow single crystals of the title compound were obtained as a minor by-product (less than 5% isolated yield) during a preparation of benzildianil from benzil and excess aniline according to the literature (Lorenz *et al.*, 1994).

### Refinement

H atoms were refined using a riding model, with aromatic C—H = 0.95 Å, tertiary C—H = 0.99 Å and U_iso_(H) = 1.2U_eq_(C).

### Crystal data

**Table:**

C_26_H_20_N_2_	*F*(000) = 760
*M**_r_* = 360.44	*D*_x_ = 1.236 Mg m^−^^3^
Monoclinic, *P*2_1_/*n*	Mo *K*α radiation, λ = 0.71073 Å
Hall symbol: -P2yn	Cell parameters from 58 reflections
*a* = 10.121 (2) Å	θ = 10–11.5°
*b* = 10.374 (2) Å	µ = 0.07 mm^−^^1^
*c* = 18.572 (4) Å	*T* = 143 K
β = 96.49 (3)°	Prism, yellow
*V* = 1937.4 (7) Å^3^	0.40 × 0.25 × 0.20 mm
*Z* = 4	

### Data collection

**Table:**

Stoe STADI4 diffractometer	*R*_int_ = 0.038
Radiation source: fine-focus sealed tube	θ_max_ = 25.0°, θ_min_ = 3.1°
graphite	*h* = −11→12
ω/θ scans	*k* = −12→6
5587 measured reflections	*l* = −22→0
3413 independent reflections	3 standard reflections every 90 min
2249 reflections with *I* > 2σ(*I*)	intensity decay: none

### Refinement

**Table:**

Refinement on *F*^2^	Primary atom site location: structure-invariant direct methods
Least-squares matrix: full	Secondary atom site location: difference Fourier map
*R*[*F*^2^ > 2σ(*F*^2^)] = 0.053	Hydrogen site location: inferred from neighbouring sites
*wR*(*F*^2^) = 0.120	H-atom parameters constrained
*S* = 1.06	*w* = 1/[σ^2^(*F*_o_^2^) + (0.0357*P*)^2^ + 0.6314*P*] where *P* = (*F*_o_^2^ + 2*F*_c_^2^)/3
3413 reflections	(Δ/σ)_max_ < 0.001
253 parameters	Δρ_max_ = 0.17 e Å^−^^3^
0 restraints	Δρ_min_ = −0.20 e Å^−^^3^

### Special details

**Table:**

Geometry. All e.s.d.'s (except the e.s.d. in the dihedral angle between two l.s. planes) are estimated using the full covariance matrix. The cell e.s.d.'s are taken into account individually in the estimation of e.s.d.'s in distances, angles and torsion angles; correlations between e.s.d.'s in cell parameters are only used when they are defined by crystal symmetry. An approximate (isotropic) treatment of cell e.s.d.'s is used for estimating e.s.d.'s involving l.s. planes.
Refinement. Refinement of *F*^2^ against ALL reflections. Weighted *R*-factors *wR* and all goodnesses of fit *S* are based on *F*^2^, conventional *R*-factors *R* are based on *F*, with *F* set to zero for negative *F*^2^. The observed criterion of *F*^2^ > σ(*F*^2^) is used only for calculating _R_factor_obs *etc*. and is not relevant to the choice of reflections for refinement. *R*-factors based on *F*^2^ are statistically about twice as large as those based on *F*, and *R*- factors based on ALL data will be even larger.

### Fractional atomic coordinates and isotropic or equivalent isotropic displacement parameters (Å^2^)

**Table:**

	*x*	*y*	*z*	*U*_iso_*/*U*_eq_	
N1	0.03731 (18)	0.21070 (18)	0.24908 (10)	0.0289 (5)	
C1	−0.0288 (2)	0.3104 (2)	0.28694 (12)	0.0288 (5)	
H1	−0.0601	0.3783	0.2508	0.035*	
C2	0.0724 (2)	0.3722 (2)	0.34379 (12)	0.0284 (5)	
N2	0.16822 (18)	0.30815 (19)	0.37782 (10)	0.0330 (5)	
C3	0.1878 (2)	0.1800 (2)	0.35655 (13)	0.0314 (6)	
C4	0.2750 (2)	0.1022 (2)	0.40081 (14)	0.0403 (6)	
H4	0.3131	0.1338	0.4465	0.048*	
C5	0.3068 (2)	−0.0197 (3)	0.37920 (14)	0.0429 (7)	
H5	0.3662	−0.0722	0.4097	0.052*	
C6	0.2513 (2)	−0.0648 (3)	0.31256 (13)	0.0389 (6)	
H6	0.2745	−0.1483	0.2971	0.047*	
C7	0.1631 (2)	0.0087 (2)	0.26827 (13)	0.0339 (6)	
H7	0.1251	−0.0246	0.2229	0.041*	
C8	0.1293 (2)	0.1322 (2)	0.28974 (12)	0.0290 (5)	
C11	−0.0053 (2)	0.1892 (2)	0.17475 (12)	0.0275 (5)	
C12	0.0861 (2)	0.1544 (2)	0.12738 (12)	0.0311 (5)	
H12	0.1769	0.1423	0.1452	0.037*	
C13	0.0448 (2)	0.1374 (2)	0.05486 (12)	0.0325 (6)	
H13	0.1078	0.1140	0.0229	0.039*	
C14	−0.0871 (2)	0.1540 (2)	0.02785 (13)	0.0378 (6)	
H14	−0.1151	0.1413	−0.0222	0.045*	
C15	−0.1772 (2)	0.1891 (2)	0.07428 (13)	0.0375 (6)	
H15	−0.2677	0.2017	0.0560	0.045*	
C16	−0.1375 (2)	0.2062 (2)	0.14755 (13)	0.0333 (6)	
H16	−0.2010	0.2297	0.1792	0.040*	
C21	−0.1490 (2)	0.2648 (2)	0.32301 (12)	0.0325 (6)	
C22	−0.1641 (3)	0.1378 (3)	0.34323 (14)	0.0440 (7)	
H22	−0.0993	0.0756	0.3338	0.053*	
C23	−0.2732 (3)	0.1008 (3)	0.37709 (16)	0.0583 (8)	
H23	−0.2823	0.0136	0.3914	0.070*	
C24	−0.3685 (3)	0.1902 (4)	0.39001 (15)	0.0589 (9)	
H24	−0.4438	0.1644	0.4128	0.071*	
C25	−0.3549 (3)	0.3163 (3)	0.37011 (14)	0.0514 (8)	
H25	−0.4202	0.3781	0.3793	0.062*	
C26	−0.2458 (2)	0.3532 (3)	0.33650 (13)	0.0405 (6)	
H26	−0.2370	0.4406	0.3225	0.049*	
C31	0.0561 (2)	0.5091 (2)	0.36335 (12)	0.0288 (5)	
C32	0.0050 (2)	0.6012 (2)	0.31318 (14)	0.0368 (6)	
H32	−0.0229	0.5759	0.2647	0.044*	
C34	0.0335 (2)	0.7668 (3)	0.40346 (15)	0.0429 (7)	
H34	0.0259	0.8544	0.4171	0.052*	
C35	0.0839 (2)	0.6766 (3)	0.45398 (14)	0.0403 (6)	
H35	0.1106	0.7023	0.5025	0.048*	
C36	0.0956 (2)	0.5495 (2)	0.43407 (13)	0.0339 (6)	
H36	0.1312	0.4884	0.4691	0.041*	
C33	−0.0058 (3)	0.7291 (2)	0.33302 (15)	0.0426 (7)	
H33	−0.0402	0.7910	0.2981	0.051*	

### Atomic displacement parameters (Å^2^)

**Table:**

	*U*^11^	*U*^22^	*U*^33^	*U*^12^	*U*^13^	*U*^23^
N1	0.0282 (10)	0.0312 (12)	0.0270 (10)	0.0036 (9)	0.0016 (8)	−0.0023 (9)
C1	0.0295 (12)	0.0277 (13)	0.0289 (12)	0.0019 (10)	0.0021 (10)	−0.0023 (11)
C2	0.0275 (12)	0.0320 (14)	0.0261 (12)	−0.0033 (11)	0.0044 (9)	0.0001 (11)
N2	0.0305 (11)	0.0336 (12)	0.0338 (11)	0.0007 (9)	−0.0008 (9)	−0.0005 (9)
C3	0.0278 (12)	0.0299 (14)	0.0354 (13)	−0.0002 (10)	−0.0006 (10)	0.0024 (11)
C4	0.0389 (14)	0.0384 (16)	0.0410 (15)	0.0018 (12)	−0.0073 (12)	−0.0018 (12)
C5	0.0398 (15)	0.0372 (16)	0.0492 (16)	0.0059 (12)	−0.0068 (12)	0.0047 (13)
C6	0.0391 (14)	0.0346 (15)	0.0436 (15)	0.0050 (12)	0.0079 (12)	0.0024 (12)
C7	0.0338 (13)	0.0328 (15)	0.0356 (13)	0.0022 (11)	0.0066 (11)	−0.0026 (11)
C8	0.0247 (12)	0.0319 (14)	0.0308 (12)	0.0004 (10)	0.0051 (10)	0.0036 (11)
C11	0.0306 (12)	0.0236 (13)	0.0281 (12)	−0.0026 (10)	0.0028 (10)	−0.0018 (10)
C12	0.0299 (12)	0.0309 (14)	0.0321 (13)	−0.0014 (11)	0.0017 (10)	−0.0013 (11)
C13	0.0397 (14)	0.0280 (14)	0.0306 (13)	0.0011 (11)	0.0069 (11)	−0.0031 (11)
C14	0.0478 (15)	0.0353 (15)	0.0289 (13)	0.0001 (12)	−0.0009 (11)	−0.0026 (11)
C15	0.0337 (14)	0.0410 (16)	0.0355 (14)	0.0014 (12)	−0.0058 (11)	−0.0034 (12)
C16	0.0288 (13)	0.0361 (15)	0.0353 (13)	0.0014 (11)	0.0054 (10)	−0.0035 (11)
C21	0.0271 (12)	0.0416 (16)	0.0282 (13)	−0.0030 (11)	0.0011 (10)	−0.0096 (11)
C22	0.0421 (15)	0.0428 (17)	0.0489 (16)	−0.0065 (13)	0.0137 (13)	−0.0051 (14)
C23	0.0592 (19)	0.058 (2)	0.0613 (19)	−0.0188 (16)	0.0202 (16)	−0.0030 (16)
C24	0.0383 (16)	0.089 (3)	0.0526 (18)	−0.0173 (17)	0.0193 (14)	−0.0163 (18)
C25	0.0333 (15)	0.076 (2)	0.0463 (17)	0.0024 (15)	0.0094 (12)	−0.0148 (16)
C26	0.0342 (14)	0.0492 (18)	0.0378 (14)	0.0043 (12)	0.0031 (11)	−0.0073 (13)
C31	0.0245 (12)	0.0293 (14)	0.0326 (13)	−0.0010 (10)	0.0033 (10)	−0.0023 (11)
C32	0.0372 (14)	0.0348 (15)	0.0374 (14)	−0.0026 (11)	−0.0008 (11)	−0.0002 (12)
C34	0.0413 (16)	0.0297 (15)	0.0584 (18)	−0.0026 (12)	0.0084 (13)	−0.0072 (13)
C35	0.0400 (14)	0.0418 (17)	0.0399 (15)	−0.0037 (12)	0.0080 (11)	−0.0116 (13)
C36	0.0315 (13)	0.0366 (15)	0.0338 (13)	0.0006 (11)	0.0043 (11)	−0.0009 (12)
C33	0.0441 (16)	0.0301 (15)	0.0523 (17)	0.0003 (12)	−0.0003 (13)	0.0049 (13)

### Geometric parameters (Å, °)

**Table:**

N1—C8	1.394 (3)	C14—H14	0.9500
N1—C11	1.417 (3)	C15—C16	1.386 (3)
N1—C1	1.455 (3)	C15—H15	0.9500
C1—C2	1.527 (3)	C16—H16	0.9500
C1—C21	1.529 (3)	C21—C22	1.383 (3)
C1—H1	1.0000	C21—C26	1.385 (3)
C2—N2	1.282 (3)	C22—C23	1.385 (4)
C2—C31	1.480 (3)	C22—H22	0.9500
N2—C3	1.408 (3)	C23—C24	1.379 (4)
C3—C4	1.394 (3)	C23—H23	0.9500
C3—C8	1.404 (3)	C24—C25	1.370 (4)
C4—C5	1.376 (3)	C24—H24	0.9500
C4—H4	0.9500	C25—C26	1.383 (4)
C5—C6	1.382 (3)	C25—H25	0.9500
C5—H5	0.9500	C26—H26	0.9500
C6—C7	1.375 (3)	C31—C32	1.392 (3)
C6—H6	0.9500	C31—C36	1.393 (3)
C7—C8	1.395 (3)	C32—C33	1.385 (3)
C7—H7	0.9500	C32—H32	0.9500
C11—C16	1.387 (3)	C34—C33	1.380 (4)
C11—C12	1.394 (3)	C34—C35	1.381 (4)
C12—C13	1.376 (3)	C34—H34	0.9500
C12—H12	0.9500	C35—C36	1.379 (3)
C13—C14	1.383 (3)	C35—H35	0.9500
C13—H13	0.9500	C36—H36	0.9500
C14—C15	1.372 (3)	C33—H33	0.9500
			
C8—N1—C11	123.18 (19)	C14—C15—C16	120.8 (2)
C8—N1—C1	117.98 (18)	C14—C15—H15	119.6
C11—N1—C1	118.46 (18)	C16—C15—H15	119.6
N1—C1—C2	108.88 (18)	C15—C16—C11	120.1 (2)
N1—C1—C21	115.09 (19)	C15—C16—H16	119.9
C2—C1—C21	109.38 (18)	C11—C16—H16	119.9
N1—C1—H1	107.7	C22—C21—C26	118.7 (2)
C2—C1—H1	107.7	C22—C21—C1	122.1 (2)
C21—C1—H1	107.7	C26—C21—C1	119.2 (2)
N2—C2—C31	118.4 (2)	C21—C22—C23	120.4 (3)
N2—C2—C1	122.3 (2)	C21—C22—H22	119.8
C31—C2—C1	119.24 (19)	C23—C22—H22	119.8
C2—N2—C3	118.30 (19)	C24—C23—C22	120.1 (3)
C4—C3—C8	119.5 (2)	C24—C23—H23	119.9
C4—C3—N2	118.8 (2)	C22—C23—H23	119.9
C8—C3—N2	121.5 (2)	C25—C24—C23	120.1 (3)
C5—C4—C3	120.9 (2)	C25—C24—H24	119.9
C5—C4—H4	119.6	C23—C24—H24	119.9
C3—C4—H4	119.6	C24—C25—C26	119.7 (3)
C4—C5—C6	119.2 (2)	C24—C25—H25	120.1
C4—C5—H5	120.4	C26—C25—H25	120.1
C6—C5—H5	120.4	C25—C26—C21	121.0 (3)
C7—C6—C5	121.3 (2)	C25—C26—H26	119.5
C7—C6—H6	119.4	C21—C26—H26	119.5
C5—C6—H6	119.4	C32—C31—C36	118.0 (2)
C6—C7—C8	120.1 (2)	C32—C31—C2	122.5 (2)
C6—C7—H7	119.9	C36—C31—C2	119.5 (2)
C8—C7—H7	119.9	C33—C32—C31	120.9 (2)
N1—C8—C7	123.5 (2)	C33—C32—H32	119.6
N1—C8—C3	117.5 (2)	C31—C32—H32	119.6
C7—C8—C3	119.0 (2)	C33—C34—C35	119.8 (2)
C16—C11—C12	119.0 (2)	C33—C34—H34	120.1
C16—C11—N1	120.7 (2)	C35—C34—H34	120.1
C12—C11—N1	120.3 (2)	C36—C35—C34	120.1 (2)
C13—C12—C11	120.1 (2)	C36—C35—H35	119.9
C13—C12—H12	120.0	C34—C35—H35	119.9
C11—C12—H12	120.0	C35—C36—C31	121.1 (2)
C12—C13—C14	120.8 (2)	C35—C36—H36	119.5
C12—C13—H13	119.6	C31—C36—H36	119.5
C14—C13—H13	119.6	C34—C33—C32	120.0 (2)
C15—C14—C13	119.2 (2)	C34—C33—H33	120.0
C15—C14—H14	120.4	C32—C33—H33	120.0
C13—C14—H14	120.4		
			
C8—N1—C1—C2	40.5 (3)	N1—C11—C12—C13	−178.0 (2)
C11—N1—C1—C2	−146.37 (19)	C11—C12—C13—C14	−0.2 (4)
C8—N1—C1—C21	−82.7 (2)	C12—C13—C14—C15	0.6 (4)
C11—N1—C1—C21	90.5 (2)	C13—C14—C15—C16	−0.8 (4)
N1—C1—C2—N2	−33.5 (3)	C14—C15—C16—C11	0.6 (4)
C21—C1—C2—N2	93.0 (3)	C12—C11—C16—C15	−0.2 (4)
N1—C1—C2—C31	149.66 (19)	N1—C11—C16—C15	177.9 (2)
C21—C1—C2—C31	−83.8 (2)	N1—C1—C21—C22	24.1 (3)
C31—C2—N2—C3	−176.7 (2)	C2—C1—C21—C22	−98.8 (3)
C1—C2—N2—C3	6.5 (3)	N1—C1—C21—C26	−156.4 (2)
C2—N2—C3—C4	−168.5 (2)	C2—C1—C21—C26	80.7 (3)
C2—N2—C3—C8	16.2 (3)	C26—C21—C22—C23	−0.8 (4)
C8—C3—C4—C5	1.4 (4)	C1—C21—C22—C23	178.7 (2)
N2—C3—C4—C5	−174.0 (2)	C21—C22—C23—C24	0.9 (4)
C3—C4—C5—C6	0.1 (4)	C22—C23—C24—C25	−0.7 (4)
C4—C5—C6—C7	−1.3 (4)	C23—C24—C25—C26	0.5 (4)
C5—C6—C7—C8	0.9 (4)	C24—C25—C26—C21	−0.5 (4)
C11—N1—C8—C7	−16.9 (3)	C22—C21—C26—C25	0.6 (4)
C1—N1—C8—C7	155.9 (2)	C1—C21—C26—C25	−178.9 (2)
C11—N1—C8—C3	164.7 (2)	N2—C2—C31—C32	148.6 (2)
C1—N1—C8—C3	−22.5 (3)	C1—C2—C31—C32	−34.5 (3)
C6—C7—C8—N1	−177.7 (2)	N2—C2—C31—C36	−30.0 (3)
C6—C7—C8—C3	0.7 (3)	C1—C2—C31—C36	146.9 (2)
C4—C3—C8—N1	176.6 (2)	C36—C31—C32—C33	0.2 (3)
N2—C3—C8—N1	−8.1 (3)	C2—C31—C32—C33	−178.4 (2)
C4—C3—C8—C7	−1.8 (3)	C33—C34—C35—C36	0.3 (4)
N2—C3—C8—C7	173.5 (2)	C34—C35—C36—C31	−0.6 (4)
C8—N1—C11—C16	139.9 (2)	C32—C31—C36—C35	0.3 (3)
C1—N1—C11—C16	−32.9 (3)	C2—C31—C36—C35	179.0 (2)
C8—N1—C11—C12	−42.1 (3)	C35—C34—C33—C32	0.2 (4)
C1—N1—C11—C12	145.1 (2)	C31—C32—C33—C34	−0.5 (4)
C16—C11—C12—C13	0.0 (3)		

## References

[bb1] Bodforss, S. (1960). *Liebigs Ann. Chem.* **633**, 66–77.

[bb2] Kehrmann, F. & Messinger, J. (1891). *Chem. Ber.* **24**, 1874–1876.

[bb3] Lorenz, V., Thiele, K.-H. & Neumüller, B. (1994). *Z. Anorg. Allg. Chem.* **620**, 691–696.

[bb4] Sannicolò, F. (1983). *J. Org. Chem.* **48**, 2924–2925.

[bb5] Sheldrick, G. M. (2008). *Acta Cryst.* A**64**, 112–122. 10.1107/S0108767307043930 18156677

[bb6] Siegfeld, M. (1892). *Chem. Ber* **25**, 2600–2601.

[bb7] Stoe & Cie (1992). *DIF4* and *REDU4* Stoe & Cie, Darmstadt, Germany.

